# The Tetanic Face

**Published:** 1846-04

**Authors:** 


*The Tetanic Face.*—The late Mr. Colles, lecturing on Tetanus, says:—

"'It is very peculiar, and if once looked at with attention, can never be forgotten. The forehead is wrinkled, both transversely and in the perpendicular direction, the eyebrows being drawn in a remarkable manner towards each other; the eyes are not fully opened; the nostrils more or less dilated; and the angles of the mouth drawn backwards and a little upwards. There is generally an expression of uneasiness, and slightly of apprehension; the mouth is not quite closed, and the teeth are seen; the body is sometimes hot and dry, but oftener the upper part is covered by perspiration, at times profuse.' Dr. Colles then remarks that, 'There is no disease which has been so often confounded with others as tetanus, although the symptoms are so well marked. For my own part, I think the countenance would, in every case, be sufficient to distinguish it from all others. I never saw but one description of face, one tetanic expression of countenance; it is the same in all cases; it is the first thing that gives the alarm, and the last symptom to depart. Even where a patient recovers, and is able to go about his business, that tetanic face remains. I believe it never leaves him.'"—*Dub. Med. Press,* Jan. 31, 1844.

There is much truth, though, we think, a little exaggeration in this. The expression of Acute Tetanus, or of Chronic, during the spasm, is certainly characteristic. Even in the intervals of the attacks, the anxiety, combined with the perspiration is peculiar. But we cannot say that we have observed any thing particular in the aspect of those who have recovered.—*Med. Chir. Review.*

